# The effect of chemotherapeutic agents on tumor vasculature in subcutaneous and orthotopic human tumor xenografts

**DOI:** 10.1186/s12885-015-1091-6

**Published:** 2015-03-10

**Authors:** Andrea S Fung, Carol Lee, Man Yu, Ian F Tannock

**Affiliations:** Department of Medical Oncology and Hematology, Princess Margaret Cancer Centre and University of Toronto, 610 University Avenue, Toronto, ON M5G 2 M9 Canada

**Keywords:** Tumor models, Orthotopic tumors, Tumor vasculature, Tumor microenvironment, Chemotherapy

## Abstract

**Background:**

The growth of solid tumors and their regrowth after treatment is dependent upon functional tumor vasculature. Some chemotherapeutic agents have shown anti-angiogenic properties but there are limited studies of the effect of chemotherapy on tumor vasculature. Here we investigate the effect of paclitaxel, 5-fluorouracil (5-FU) and doxorubicin on tumor vasculature in subcutaneous and orthotopic xenografts in mice.

**Methods:**

The vascular density and percentage of functional blood vessels were evaluated in subcutaneous A431 human vulvar cancer xenografts, and in subcutaneous and orthotopic MCF-7 human breast cancer xenografts, following single doses of paclitaxel, 5-FU or doxorubicin.

**Results:**

There was no significant difference in total (CD31+) blood vessels between untreated ectopic and orthotopic MCF-7 tumors, but there was a significantly lower proportion of functional blood vessels in orthotopic tumors. After paclitaxel treatment, there was a decrease in functional tumor vasculature in A431 subcutaneous xenografts, followed by a subsequent rebound. There was a significant decrease in total vascular density on day 12 in A431 tumors following 5-FU or doxorubicin treatment, but no change in the percentage of functional vessels. An increase in functional blood vessels or percentage of functional vasculature was noted in MCF-7 subcutaneous and orthotopic xenografts following chemotherapy treatment.

**Conclusions:**

There are differences in the vasculature and microenvironment of ectopic and orthotopic xenografts in mice. Anti-tumor effects of chemotherapy may be due, in part, to effects on tumor vasculature and may vary in different tumor models.

## Background

The presence of functional vasculature within solid tumors is required to provide sufficient nutrients and oxygenation to tumor cells, and is therefore essential for the growth of solid tumors and for their repopulation after treatment [1]. Adequate drug distribution within solid tumors is also dependent on functional vasculature. Therefore, evaluating the ability of various chemotherapeutic treatments to alter tumor vasculature has important implications for understanding the effects of treatment.

Angiogenesis, the formation of new blood vessels, occurs through a delicate balance between pro- and anti-angiogenic factors, such as VEGF and thrombospondin-1, respectively [2,3]. Given the dependence of tumor growth on tumor vascularity, numerous studies have focused on targeting the process of angiogenesis. Drugs targeted against the vascular endothelial growth factor (VEGF) pathway, such as bevacizumab, are being utilized in the clinic [4]. However, data suggest that other anti-cancer agents, including chemotherapy and targeted agents such as EGFR inhibitors, might also have effects to decrease the quantity or functionality of tumor vasculature [5-10].

A few studies have shown that taxanes, such as paclitaxel and docetaxel, can inhibit endothelial cell proliferation *in vitro*, and have vascular disrupting properties *in vivo* resulting in decreased vascular density within treated tumors [5-8]. Shaked *et al*. showed that paclitaxel, docetaxel, and 5-FU all caused a decrease in microvascular density and a corresponding increase in the recruitment of circulating endothelial progenitors (CEPs), which might contribute to vascular rebound following treatment, whereas, gemcitabine, cisplatin, and doxorubicin did not have an effect on vascular density or circulating endothelial progenitors [9]. Metronomic chemotherapy (i.e. chemotherapy administered at lower doses at more frequent intervals) has also shown antiangiogenic properties through increased endothelial cell apoptosis [11-17].

Various tumor models are utilized to investigate the efficacy of anti-cancer therapies. Ectopic tumor xenografts are often used to assess antitumor activity of cytotoxic or cytostatic agents due to the reproducibility and ease of access of this tumor model when evaluating tumor growth [18]. However, studies suggest that orthotopic tumors (i.e. tumor cells implanted at the site of origin) are more similar to clinical tumors due to the establishment of a heterogeneous tumor microenvironment, the expression of biologically relevant growth factor receptors and proteins, and the metastatic potential of tumor cells to spread to distant sites, thereby reflecting the natural course of clinical cancers [18,19]. Studies by Fidler and colleagues have shown that the expression of multidrug resistance genes and proteins can differ depending on the organ environment, thereby altering the efficacy of chemotherapy against tumors implanted at different organ sites [20-22]. These studies highlight the effect of the organ environment on tumor growth and response to therapy; therefore, it is important to determine whether different organ sites might also impact the tumor vasculature and microenvironment.

The present study aims to investigate whether the commonly used anticancer drugs paclitaxel, 5-FU, and doxorubicin modify the functional vasculature of subcutaneous A431 xenografts, and of subcutaneous or orthotopic MCF-7 xenografts.

## Methods

### Cell lines

Experiments were performed using the vulvar epidermoid carcinoma cell line A431, and the breast carcinoma cell line MCF-7. All cells were purchased from the American Type Culture Collection (ATCC; Manassas, VA). A431 cells were maintained in Dulbecco’s Modified Eagle’s Medium supplemented with 10% fetal bovine serum (FBS; Hyclone, Logan, UT). MCF-7 cells were grown in α-MEM with 10% FBS. All media was obtained from the hospital media facility. Cells were grown in a humidified atmosphere of 95% air and 5% CO_2_ at 37°C. Routine tests to confirm cell line origin, and to exclude mycoplasma were performed.

### Drugs and reagents

Paclitaxel, 5-FU and doxorubicin were standard clinical formulations purchased from the hospital pharmacy, and were diluted in PBS. DiOC7 was purchased from AnaSpec Inc. (San Jose, CA) and a stock solution (2.5 mg/mL) was made by dissolving DiOC7 powder in DMSO. The stock was diluted 1:10 in PBS and 10% Solutol HS 15.

### Effect of paclitaxel, 5-FU and doxorubicin on tumor vasculature

Female athymic nude mice (4 to 6 weeks old) (Harlan Sprague-Dawley (HSD), Madison, WI) were injected subcutaneously on both flanks with 1×10^6^ A431 cells or 4×10^6^ MCF-7 cells per side to generate xenografts. Prior to injection of MCF-7 cells, mice were implanted with 17β estradiol tablets (60 day release; Innovative Research of America, Sarasota, FL). For orthotopic tumors, 1×10^6^ MCF-7 cells (suspended in Matrigel) were injected into the mammary fat pads of 4-6 week old female athymic nude mice (HSD). Two perpendicular diameters were measured with a calliper and when tumors grew to a diameter of 5-8 mm, mice were treated with a single dose of paclitaxel (25 mg/kg i.p.), 5-FU (100 mg/kg i.p.) or doxorubicin (8 mg/kg i.v.).

Tumor samples were taken on days 0, 2, 4, 8 and 12 following administration of the chemotherapy drug. The perfusion marker DiOC7 (1 mg/kg) was injected intravenously 1 minute prior to killing the mice. Tumors were excised, immersed in OCT compound and frozen in liquid nitrogen. Tumors were cut into 10 μm sections and imaged using an Olympus BX50 fluorescence microscope.

Tumor sections were first imaged for the perfusion marker DiOC7 using a FITC filter set. Sections were then stained for blood vessels using antibodies specific for the endothelial cell marker CD31 [rat anti-CD31 primary antibody (1:100); BD Biosciences, and Cy3-conjugated goat anti-rat IgG secondary antibody (1:400)]. Tumor sections were imaged for CD31 using the Cy3 (530-560 nm excitation/573-647 nm emission) filter set.

### Image analysis and quantification

Microscope images of tumor vasculature were quantified using Media Cybernetics Image Pro PLUS software. A threshold was used to select pixels occupied by blood vessels, as represented by CD31 staining, and the image was binarised by setting these blood vessel regions to white (pixel value 255) and background pixels to black (pixel value 0) to form a “mask” of positive CD31 staining. Using Image Pro’s Count/Size tool, objects with a pixel intensity of 255 (i.e. CD31-positive) were counted in each tumor section. The tumor area was measured by drawing an ‘Area of Interest’ around tumor regions on the image (excluding areas of necrosis or artefacts) using Image Pro’s calibrated area measurement tool. The mean number of total blood vessels per tumor area was calculated. A similar method was used to evaluate functional blood vessels: the total number of objects in DiOC7 binarised images was counted and the number of DiOC7-positive objects was divided by the number of CD31-positive objects to provide an estimate of the proportion of functional vessels in each tumor section.

### Statistical analysis

For analysis of total and functional tumor vasculature, t-tests were performed to determine significant differences between groups. P < 0.05 was used to indicate statistical significance; all tests were 2-sided.

### Animal ethics statement

Animal experiments were carried out using protocol (AUP #1232.15) approved by the University Health Network (UHN) Animal Care Committee under the guidelines of the Canadian Council on Animal Care.

## Results

### Effect of chemotherapy on tumor size in A431 and MCF-7 xenografts

Mice were treated with a single dose of paclitaxel, doxorubicin, or 5-FU. Dose response studies were previously completed in the laboratory, and the dose of each chemotherapy agent was chosen based on optimal anti-tumor effect with minimal toxicity as determined by measurement of body weight (data not shown). Tumor size was measured by calculating the pixel area occupied by the tumor (Table 1). Untreated (Day 0) MCF-7 xenografts were larger in size compared to A431 xenografts. There was no significant difference in the tumor size of untreated ectopic and orthotopic tumors. The tumor area of Day 12 A431 xenografts treated with paclitaxel was significantly smaller than control tumors (P = 0.03); however, there was no significant change in tumor size in A431 xenografts treated with doxorubicin or 5-FU. There was no difference in tumor area of ectopic and subcutaneous MCF-7 xenografts treated with paclitaxel or doxorubicin (all time points). Subcutaneous MCF-7 xenografts taken on Day 4 following 5-FU treatment had a significantly smaller tumor area compared to control tumors (P = 0.001); there was no difference in tumor size in ectopic MCF-7 xenografts treated with 5-FU.

**Table 1:** **Tumor area as measured by number of pixels (x10** ^**7**^ **) for A431 xenografts, and ectopic or orthotopic MCF-7 xenografts taken on Day 0 (untreated), 2, 4, 8, or 12 following a single dose of paclitaxel (25 mg/kg, i.p.), doxorubicin (DOX 8 mg/kg, i.v.), or 5-FU (100 mg/kg, i.p.)**

Day	A431			MCF-7 Paclitaxel		MCF-7 DOX		MCF-7 5-FU	
	Paclitaxel	DOX	5-FU	SC	Ortho	SC	Ortho	SC	Ortho
**0**	1.18 (0.27)	2.29 (0.25)	2.29 (0.25)	4.79 (0.65)	7.40 (4.60)	4.79 (0.65)	7.40 (4.60)	4.79 (0.65)	7.40 (4.60)
**2**	______	2.43 (0.38)	1.96 (0.19)	______	3.25 (1.41)	6.99 (0.91)	2.53 (0.55)	7.27 (1.16)	5.34 (1.24)
**4**	1.67 (0.16)	2.70 (0.49)	2.71 (0.49)	3.94 (0.61)	5.04 (1.41)	4.86 (1.33)	3.29 (---)	1.27 (0.53)*	4.49 (2.08)
**8**	0.99 (0.15)	2.42 (0.36)	1.92 (0.32)	3.78 (0.47)	5.20 (0.80)	3.39 (0.63)	4.06 (1.35)	5.11 (1.17)	5.85 (0.76)
**12**	0.47 (0.06)*	2.11 (0.47)	1.27 (0.37)	4.57 (0.33)	2.05 (0.82)	3.17 (0.65)	______	3.34 (0.85)	7.48 (0.83)

Symbols (*) represent statistically significant differences in tumor area compared to control tumors (P < 0.05).

### Effect of various chemotherapy drugs on tumor vasculature in A431 xenografts

**Figure 1:**
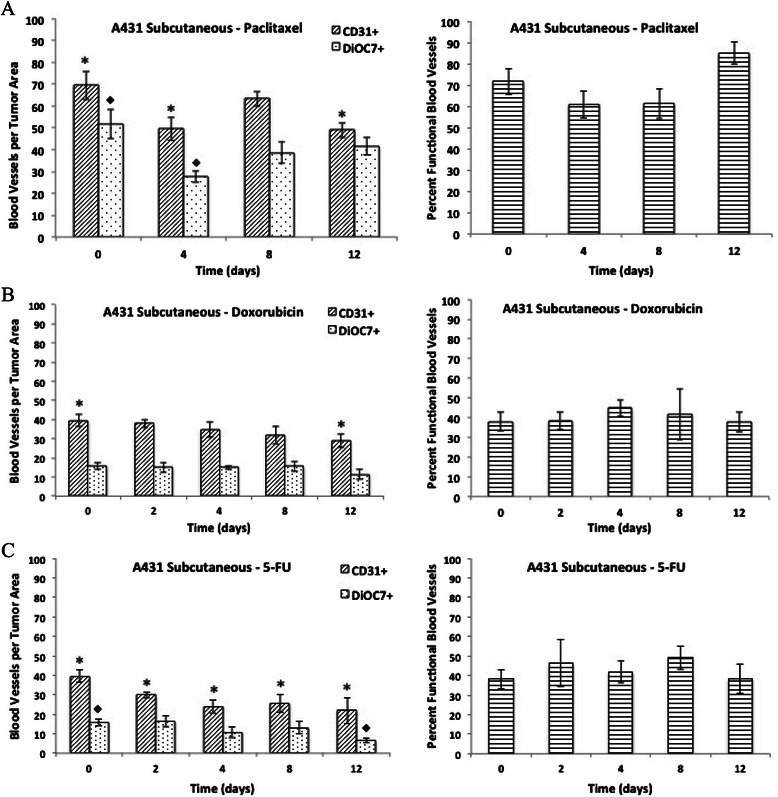
**The number of total (CD31+) and functional (DiOC7+) blood vessels per tumor area (left panels), and the percentage of functional blood vessels (right panels) present on Day 0, 2, 4, 8, and 12 in A431 xenografts following a single dose of A) paclitaxel (25 mg/kg, i.p.), B) doxorubicin (8 mg/kg, i.v.), or C) 5-FU (100 mg/kg, i.p.).** Bars represent the mean of 5-6 tumors; error bars represent standard error of the mean (SEM). Symbols represent statistical significance for total (*), functional (♦), and percent functional (+) blood vessels as compared to control (Day 0) tumors.

There was a significant decrease in the total (CD31+) and functional (DiOC7+) blood vessels on days 4 and 12, and on days 4, 8, and 12, respectively, following a single dose of paclitaxel (P < 0.05). A decrease in the percentage of functional tumor vasculature was also noted in A431 xenografts on days 4 and 8 following a single dose of paclitaxel compared to untreated (day 0) tumors; this was followed by a subsequent increase in the percentage of functional blood vessels back to pre-treatment levels by day 12 (Figure 1A).

There was a significant decrease in the total (CD31+) blood vessels per unit area in day 12 tumors compared to untreated tumors following doxorubicin treatment (Figure 1B, P = 0.04). A significant decrease in vascular density was also noted in A431 tumors taken on day 2, 4, 8, and 12 following treatment with 5-FU when compared to controls (Figure 1C, P < 0.05). However, there was no significant change in the percentage of functional blood vessels present in A431 xenografts treated with 5-FU (100 mg/kg) or doxorubicin (8 mg/kg) (Figure 1B and C, P > 0.05).

### Differences in tumor vasculature in ectopic versus orthotopic MCF-7 tumors

**Figure 2:**
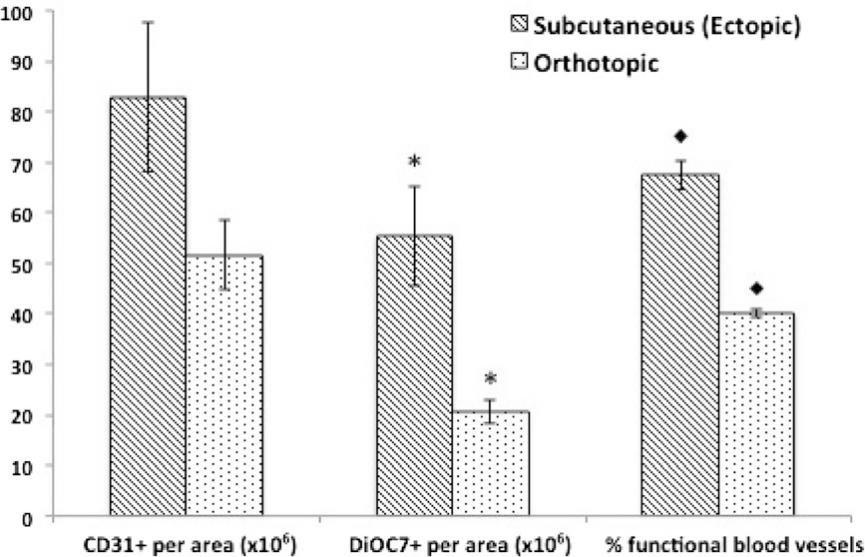
**The number of total (CD31+) and functional (DiOC7+) blood vessels (per tumor area), and the percentage of functional blood vessels present in untreated MCF-7 subcutaneous or orthotopic xenografts.** Bars represent the mean of 2-12 tumors; error bars represent standard error of the mean (SEM). Scale – numeric values represent number of blood vessels per unit area (x10^6^) for the left and middle panels, and percent functional blood vessels for the right panel. Symbols represent statistical significance between subcutaneous and orthotopic tumors for functional (*) and percent functional (♦) blood vessels.

**Figure 3:**
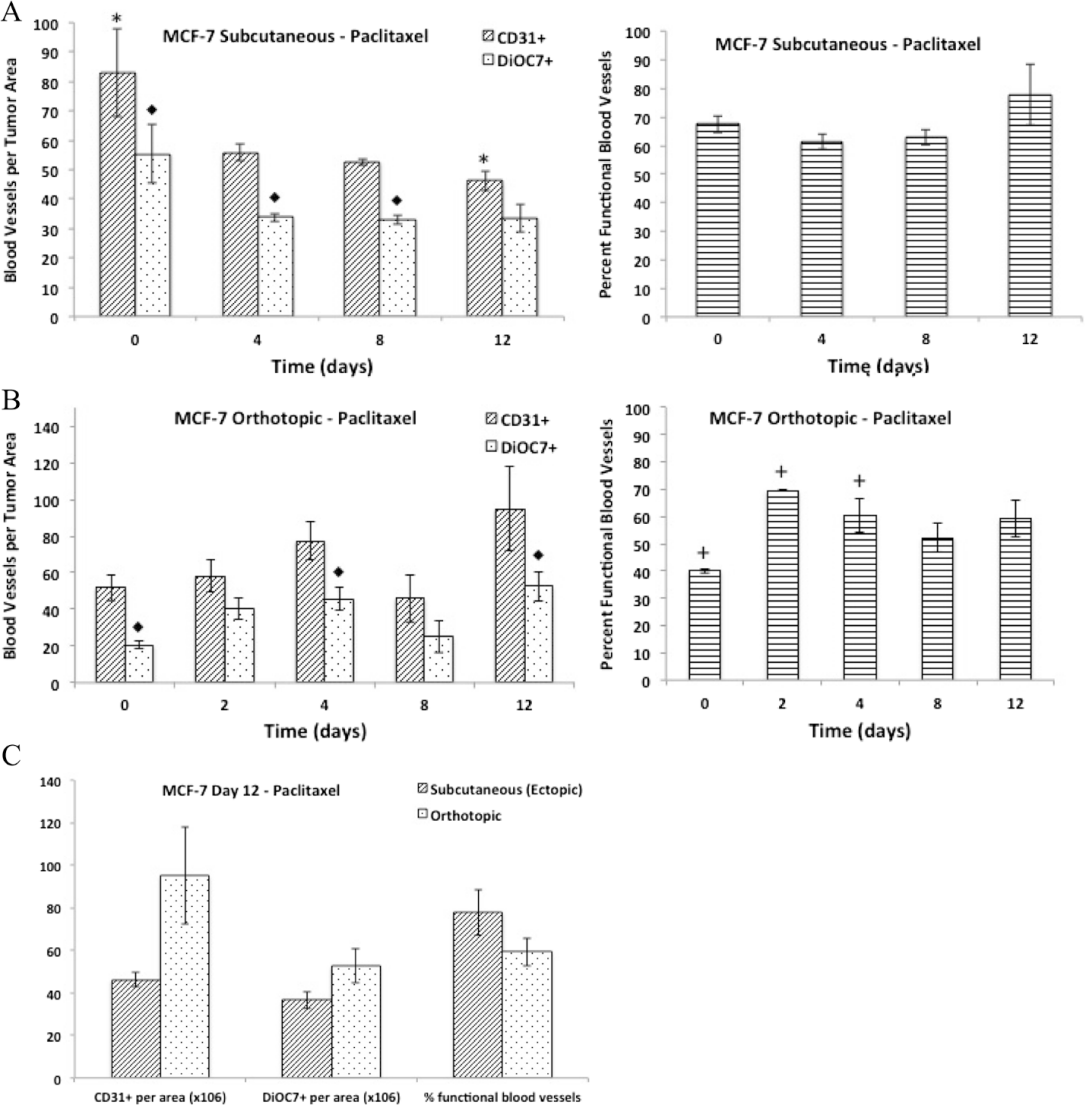
**Effect of paclitaxel on tumor vasculature in MCF-7 xenografts.** The number of total (CD31+) and functional (DiOC7+) blood vessels per tumor area (left panels), and the percentage of functional blood vessels (right panels) present on Day 0, 2, 4, 8, and 12 in MCF-7 **A)** subcutaneous xenografts or **B)** orthotopic xenografts following a single dose of paclitaxel (25 mg/kg, administered intraperitoneally). **C)** The number of total (CD31+) and functional (DiOC7+) blood vessels (per tumor area), and the percentage of functional blood vessels present in Day 12 MCF-7 subcutaneous or orthotopic xenografts. Bars represent the mean of 2-12 tumors; error bars represent standard error of the mean (SEM). Symbols represent statistical significance for total (*), functional (♦) and percent functional (+) blood vessels as compared to control (Day 0) tumors.

**Figure 4:**
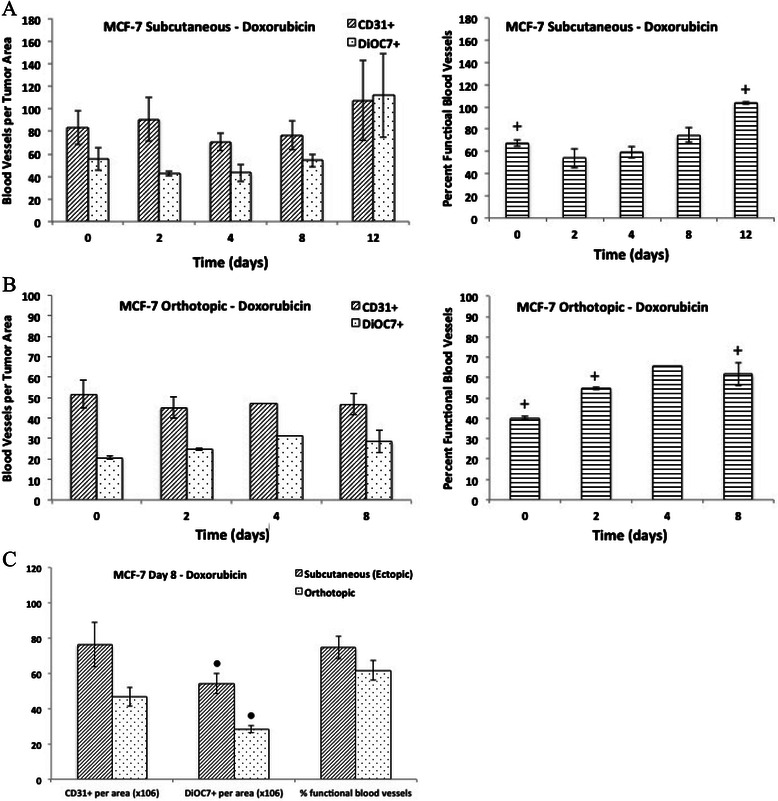
**Effect of doxorubicin on tumor vasculature in MCF-7 xenografts.** The number of total (CD31+) and functional (DiOC7+) blood vessels per tumor area (left panels), and the percentage of functional blood vessels (right panels) present on Day 0, 2, 4, 8, and 12 in MCF-7 **A)** subcutaneous xenografts or **B)** orthotopic xenografts following a single dose of doxorubicin (8 mg/kg, administered intravenously). **C)** The number of total (CD31+) and functional (DiOC7+) blood vessels (per tumor area), and the percentage of functional blood vessels present in Day 12 MCF-7 subcutaneous or orthotopic xenografts. Bars represent the mean of 2-6 tumors; error bars represent standard error of the mean (SEM). Symbols represent statistical significance for percent functional blood vessels (+) as compared to control (Day 0) tumors, and significance between subcutaneous and orthotopic tumors (●).

**Figure 5:**
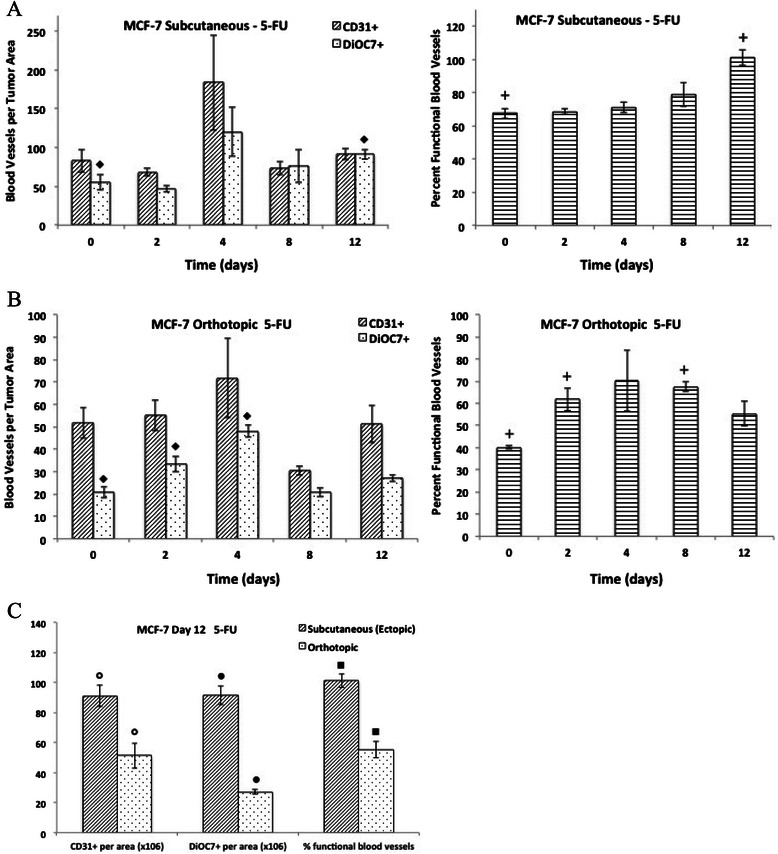
**Effect of 5-fluorouracil on tumor vasculature in MCF-7 xenografts.** The number of total (CD31+) and functional (DiOC7+) blood vessels per tumor area (left panels), and the percentage of functional blood vessels (right panels) present on Day 0, 2, 4, 8, and 12 in MCF-7 **A)** subcutaneous xenografts or **B)** orthotopic xenografts following a single dose of 5-fluorouracil, 5-FU (100 mg/kg, administered intraperitoneally). **C)** The number of total (CD31+) and functional (DiOC7+) blood vessels (per tumor area), and the percentage of functional blood vessels present in Day 12 MCF-7 subcutaneous or orthotopic xenografts. Bars represent the mean of 2-6 tumors; error bars represent standard error of the mean (SEM). Symbols represent statistical significance for functional and percent functional (+) blood vessels as compared to control (Day 0) tumors, and significance between subcutaneous and orthotopic tumors.

The vascular density (CD31+ blood vessels per tumor area) was not significantly different between ectopic and orthotopic MCF-7 tumors. However, there was a significantly lower number of functional (DiOC7+) blood vessels, and a lower percentage of functional vasculature in untreated orthotopic MCF-7 tumors when compared to ectopic tumors grown subcutaneously (Figure 2, P < 0.05). Ectopic MCF-7 tumors taken on Day 8 or 12 following chemotherapy treatment were compared to orthotopic MCF-7 tumors from the same time point to determine if there were any differences in vascular density, number of functional vessels, or percentage of functional blood vessels. There was no difference in tumor vasculature between ectopic and orthotopic MCF-7 tumors treated with paclitaxel (Figure 3C). A significantly lower number of functional blood vessels (DiOC7+) were noted in orthotopic tumors on Day 8 following doxorubicin treatment when compared to ectopic tumors (Figure 4C, P = 0.008). Orthotopic MCF-7 xenografts treated with 5-FU had a significantly lower number of total (CD31+) and functional (DiOC7+) blood vessels, and a lower percentage of functional vessels (Figure 5C, P < 0.01) on Day 12 after treatment.

### Effect of paclitaxel on tumor vasculature in subcutaneous (ectopic) and orthotopic MCF-7 xenografts

A decrease in the number of functional blood vessels (DiOC7+ vessels per unit area) was noted on day 4 and 8 following treatment of subcutaneous (ectopic) MCF-7 xenografts with a single dose of paclitaxel (Figure 3A, P < 0.05) and there was a significant decrease in total (CD31+) blood vessels on day 12 compared to control tumors (P = 0.03). There was no significant difference in the percentage of functional vasculature in ectopic MCF-7 xenografts taken on days 2-12 following a single dose of paclitaxel when compared to untreated tumors (Figure 3A, P > 0.05). Conversely, there was an increase in the number of functional (DiOC7+) blood vessels and the percentage of functional vasculature in orthotopic MCF-7 tumors on day 4 compared to control (Figure 3B, P < 0.05); however, there was no significant change in the total (CD31+) tumor vasculature with paclitaxel treatment in orthotopic tumors.

### Effect of doxorubicin on tumor vasculature in subcutaneous and orthotopic MCF-7 xenografts

There was no significant difference in the total (CD31+) or functional (DiOC7+) vascular density in MCF-7 ectopic or orthotopic tumors following doxorubicin treatment. On day 2 following doxorubicin treatment there was a decrease in the percentage of functional blood vessels in subcutaneous MCF-7 xenografts, although this was not a significant change (P > 0.05); there was a subsequent rebound in the functional vasculature back to control levels by day 8 (Figure 4A). There was a significant increase in the percentage of functional tumor vasculature in both day 12 ectopic and day 8 orthotopic tumors, respectively, when compared to controls (Figure 4A and B, P < 0.01 and P = 0.04, respectively).

### Effect of 5-fluorouracil (5-FU) on tumor vasculature in subcutaneous and orthotopic MCF-7 xenografts

There was a significant increase in the functional (DiOC7+) vascular density and percentage of functional blood vessels noted in subcutaneous MCF-7 xenografts on day 12 following 5-FU treatment compared to untreated tumors (Figure 5A, P < 0.01 respectively); however, there was no difference in total vascular density (CD31+) following 5-FU treatment (Figure 5A, P > 0.05). In the orthotopic MCF-7 xenografts, there was a significant increase in the functional (DiOC7+) blood vessels in tumors taken on days 2 and 4 compared to controls, as well as an increase in the percentage of functional vasculature noted in day 2 and 8 tumors compared to controls (Figure 5B, P < 0.05); there was no significant change in the total (CD31+) vascular density following 5-FU treatment of orthotopic MCF-7 tumors (Figure 5B, P > 0.05).

## Discussion

The growth of solid tumors and their repopulation after treatment are dependent upon functional tumor vasculature; however, the delivery of anti-cancer therapies also requires proper vascular architecture within a solid tumor [23-27]. Given the complex interactions of chemotherapy with the tumor microenvironment and tumor vasculature, we aimed to investigate the effects of paclitaxel, doxorubicin and 5-fluorouracil in different tumor models, including a comparison of subcutaneous and orthotopic MCF-7 xenografts.

Tumor models such as subcutaneous and orthotopic xenografts grown in nude mice have long been used in the investigation of the efficacy of anti-cancer agents. Studies have highlighted the importance of organ specific environments in the development of biologically heterogeneous tumors that more closely mimic the clinical progression of solid tumors and possess a similar metastatic potential [18,19]. Furthermore, tumors grown orthotopically have been shown to respond differently to anti-cancer agents compared to subcutaneous tumors [20-22]. However, few studies have investigated differences in the tumor microenvironment between ectopic and orthotropic xenografts and there are limited data on the effect of chemotherapeutic agents to alter tumor vasculature in these models.

We did not find a marked difference in total vascular density in untreated ectopic versus orthotopic MCF-7 tumors but there were fewer functional blood vessels (DiOC7+ per tumor area) in orthotopic MCF-7 tumors (Figure 2). A study by Ho *et al*. showed higher vascular density in orthotopic breast tumors compared to subcutaneous tumors of similar size [28], but the CD31 endothelial marker was utilized in their study, which makes it difficult to ascertain whether there was a similar difference in functional vasculature. Our study utilizes the perfusion marker DiOC7 in addition to CD31 to evaluate changes in the functional blood vessels, and highlights the importance of quantifying both total and functional vasculature.

Our observation of an initial decrease in the percentage of functional blood vessels following treatment of subcutaneous A431 and MCF-7 xenografts with paclitaxel (Figures 1A and 3A) agrees with previous studies showing decreased vascular density following treatment of experimental tumors with taxanes [5-8]. Interestingly, we found no significant effect of paclitaxel on the functional tumor vasculature in orthotopic tumors (Figure 3B).

A series of studies by Fidler and colleagues showed that colon carcinoma tumors grown subcutaneously had a greater anti-tumor response to doxorubicin compared to orthotopic tumors, whereas they showed a comparable response to 5-FU. Analysis of different organ sites showed differential expression of *mdr* genes, which influence response to doxorubicin but not to 5-FU [18,20-22]. In the present study, subcutaneous and orthotopic MCF-7 xenografts treated with either doxorubicin or 5-FU, and orthotopic tumors treated with paclitaxel showed a delayed increase in the percentage of functional blood vessels despite similar tumor sizes in treated tumors compared to controls (Table 1; Figures 3B, 4 and 5). Previous studies have demonstrated anti-angiogenic properties of taxanes, through targeting of cycling endothelial cells [5-8]; however, Shaked *et al*. showed that chemotherapeutic agents such as taxanes and 5-FU also initiate a systemic response leading to the recruitment of circulating endothelial progenitors (CEPs), which stimulate the process of angiogenesis [9]. Increases in functional vasculature noted in our study following chemotherapy treatment in MCF-7 tumors could be related to recruitment of CEPs or to changes in the tumor microenvironment, including changes in interstitial fluid pressure or normalization of tumor vasculature following chemotherapy [29,30]. Interestingly, there was a significantly lower number of total (CD31+) and functional (DiOC7+) blood vessels, as well as a lower percentage of functional vasculature, in orthotopic MCF-7 tumors taken on Day 12 following 5-FU treatment as compared to ectopic (subcutaneous) MCF-7 xenografts (Figure 5C, P < 0.01). Perhaps differences in the tumor microenvironment, recruitment of CEPs, or differential gene expression between different organ sites in which orthotopic and ectopic tumors are grown, might have contributed to the lack of rebound in tumor vasculature noted in orthotopic tumors following 5-FU treatment.

A strength and novelty of the current study is that both total and functional vasculature were characterized through the utilization of a flow marker in addition to immunohistochemical staining for total (CD31+) blood vessels in order to compare the differences in vasculature in different tumor models (ectopic versus orthotopic xenografts) following treatment with various chemotherapy agents. We observed different effects of chemotherapy on total and functional vasculature, thus emphasizing the importance of analyzing changes in functional vasculature. The current study also highlights the importance of the organ environment when choosing tumor models. A major weakness of the present study is that blood vessels in transplanted tumors, independent of site of transplantation, are derived from the host and may not reflect those in a spontaneous tumor. However, orthotopic tumors appear to more closely represent the clinical course of cancer progression when compared to ectopic tumors, and our data suggest that utilization of orthotopic tumor models might be more appropriate when using transplanted tumors in determining clinical effects of anti-cancer treatments.

## Conclusions

The present study shows that there are differences in the vasculature and tumor microenvironment of ectopic and orthotopic xenografts in mice. Anti-tumor effects of chemotherapy may be due, in part, to effects on tumor vasculature and may vary in different tumor models.
